# Three Conformations of Polyglutamic Acid Monitored by Vibrational Optical Activity

**DOI:** 10.1021/acs.analchem.5c05531

**Published:** 2025-12-12

**Authors:** Andrii S. Kurochka, Jana Hudecová, Josef Kapitán, Jiří Kessler, Petr Bouř

**Affiliations:** † Institute of Organic Chemistry and Biochemistry, 89220 Academy of Sciences, Flemingovo Náměstí 2, 16610 Prague, Czech Republic; ‡ Department of Optics, Palacký University Olomouc, 17, Listopadu 12, 77900 Olomouc, Czech Republic

## Abstract

Polyglutamic acid (PGA) is an excellent model system to study peptide and protein folding. Its conformation in solution can be conveniently studied by vibrational optical activity. To better understand the behavior of the molecule in different protonation states and advance the spectroscopic methodology, we obtained infrared (IR), vibrational circular dichroism (VCD), Raman, and Raman optical activity (ROA) spectra of various PGA forms and interpreted them on the basis of molecular dynamics (MD) and density functional theory (DFT) computations. The spectra include the ROA of PGA fibrils, which have been rather unexplored so far. The fibrils provided a distinct ROA pattern, which could be verified by the measurement of both enantiomers. Advancements in the use of vibrational spectroscopy for amyloid fibrils may contribute to the understanding of the biological role of these protein forms, often accompanying neurodegenerative diseases. The computations provided a reliable link between the spectral shapes and molecular geometry, and the simulated spectra reproduced the most important experimental features, although band-to-band simulations of the fibril vibrational optical activity remain challenging. The results nevertheless clearly show that vibrational optical activity combined with spectral simulations appears as a handy tool to study the geometry of proteins, including their aggregates.

## Graphical abstract

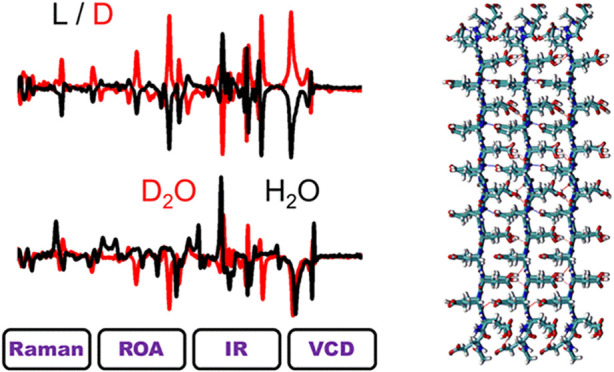

## Introduction

In the present study, we “revisit” polyglutamic acid (PGA), which has long served as a versatile model to understand protein folding and aggregation. It is commercially available in both enantiomeric forms, and its conformation can be easily induced by the acidity of aqueous solutions. At neutral pH, a disordered state is present, which changes to α-helix at lower pH.^1^ Under more acidic conditions (pH < 4.5), fully protonated PGA self-assembles into β-sheet-rich fibrils, with a sigmoidal kinetics characteristic of amyloid formation.^2^ These fibrils are stabilized by a bifurcated hydrogen bonding, involving both backbone amide groups and side-chain carboxyls.^3^

Vibrational optical activity (VOA) is one of the limited number of methods suitable for monitoring peptide and protein structures in solutions.^4^ It has provided immensely useful results for the PGA in the past. For example, vibrational circular dichroism (VCD) spectra revealed that the so-called “random coil” or “disordered” forms of PGA and other peptides and proteins are, to a large extent, formed by left-handed helical segments adopting the polyproline II (PPII) conformation.^5^ The complexation of PGA with porphyrins could be studied by VCD as well.^6^ Temperature and salt concentrations were shown to affect the PGA secondary structure.^7^ More recently, the ability of PGA to make fibril aggregates attracted attention as a model for studying the fibril formation implicated in human diseases (Alzheimer’s, Parkinson’s, type-2 diabetes).^8^ A modified PGA was used to study the fibril formation kinetics.^9^ Other fibril properties could be revealed using the IR and VCD spectra of related peptides.^10,11^ Fibrillar PGA and other peptides and proteins often give a VCD signal with enhanced intensity that is particularly convenient for the detection of the fibril formation and helicity.^12^ Only lately have similar enhancements been observed for some protein fibrils in ROA spectra.^13^

However, the interpretation of these spectra is often difficult. Molecular modeling and simulations are often necessary to fully understand experimental data. Theoretical models have indicated that the β_2_ structure^2^ is present in fibril aggregates.^11^ VCD studies of ^13^C-isotopically labeled PGA decapeptides combined with density functional theory (DFT) computations indicated antiparallel β-sheet packing in the fibrils.^14^ α-Helical PGA served as a computational model to explore the dependence of ROA spectra on the backbone torsion angles.^15^ In this context, the Cartesian coordinate-based transfer (CCT) of vibrational atomic properties^16,17^ appeared quite useful, allowing the faithful reproduction of the VCD^18^ and ROA^19^ spectra of globular proteins or α-synuclein forms containing hundreds of atoms.^20^

The Raman and ROA spectra of PGA solutions have been reported for pH 12.6 and 4.8, where the polymer exists in a disordered and partially α-helical state, respectively.^21^ The α-helical state was found to likely exist also in DMSO,^22^ and ROA studies contributed to the understanding of peptide conformational transitions.^23,24^ However, to the best of our knowledge, reliable ROA spectra of PGA fibrils have not been reported so far due to difficulties in measurement. In particular, the laser light may be reflected from the fibril surface without the possibility of being Raman-scattered, or the light may be polarized in a way that obscures the true ROA signal.

Previous efforts thus call for improved experimental methodologies as well as more accurate simulations, effectively increasing the spatial resolution of vibrational spectroscopies. Therefore, we acquired a comprehensive set of Raman, ROA, IR, and VCD spectra of PGA in three conformational states. This allowed us to develop and verify a computational methodology to simulate the spectra and other PGA properties. For example, the reliability of the ROA and VCD spectra could be checked by measuring both the l- and d-enantiomers, and the vibrational band assignments could be verified by comparing the natural and deuterated polypeptides.

It appeared that the multiscale molecular dynamics/DFT computations could very well reproduce the experimental data. Some inconsistencies remain in the modeling of fibrils due to the lack of more accurate geometry models and possibly due to incomplete light-scattering theories.^25^ These results nevertheless illustrate the immense possibilities of VOA for probing protein folding, structure, solvation, and various levels of chirality.

## Results and Discussion

### VOA Spectra of Polyglutamic Conformations

The ability of the vibrational spectra to faithfully reflect the conformation and protonation states of the molecule can be seen in Figure 1. The experimental ROA, Raman, VCD, and IR results are shown for the three pH values. In the IR and Raman spectra, the degree of protonation affects the relative intensities of the asymmetric C=O stretching (∼1565 cm^–1^) and C=O stretching (∼1740 cm^–1^) bands in the COO^–^ and COOH/COOD groups. At pH 7, the side chains are deprotonated, and the resultant disordered conformation is close to the polyproline II helix.^5^ This form is stabilized by the repulsion of the side-chain charges and their interaction with the polar aqueous environment. In a brief interval of pH (4.2–5.5), partial protonation allows for the folding into the α-helix. Below pH 4.2, the side chains are fully protonated, and the resultant β-sheet-based aggregates make the solution opalescent.

**Figure 1:**
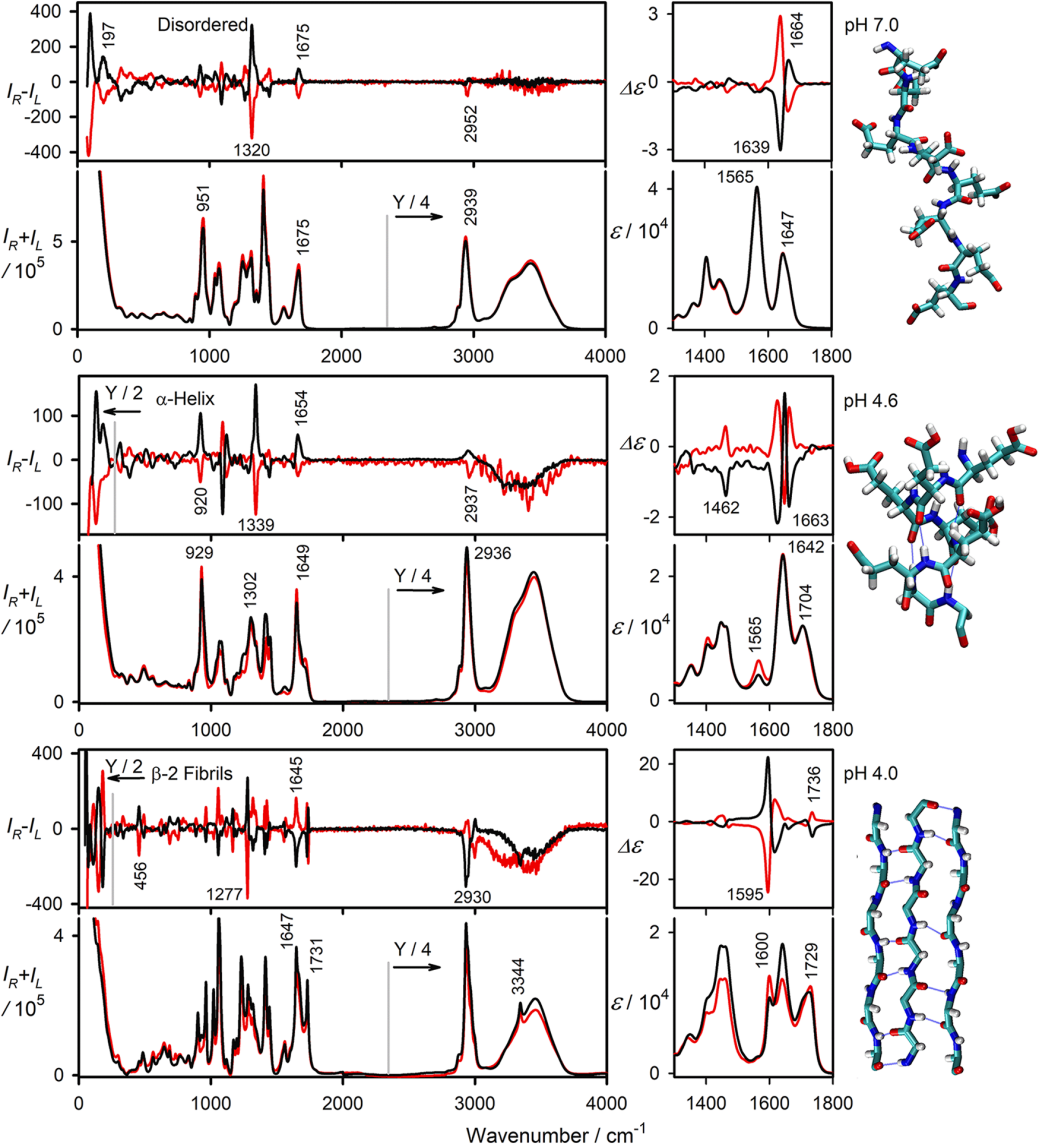
Experimental ROA (*I*_R_ – *I*_L_), Raman (*I*_R_ + *I*_L_), VCD (Δε), and IR (ε) spectra of L (black) and D (red) PGA enantiomers, measured in H_2_O (ROA, Raman, 75 mg/mL), D_2_O (VCD, IR, 23 mg/mL), and at three pH values. Note that the Raman intensity within 2400–4000 cm^–1^ was divided by 4, and the ROA intensity of the α-helix and fibrils within 50–250 cm^–1^ was divided by 2 as indicated. Examples of relevant geometries obtained from MD simulations are shown on the right.

For all chiroptical spectra, the “mirror symmetry” of the L- and D-enantiomers confirms the reliability of the measurement. Some artifacts remain mostly in the OH stretching region (∼3100–3700 cm^–1^) due to the large Raman scattering. Due to nonadditive effects,^26^ it was also difficult to fully subtract the water Raman signal. Since water solutions are problematic for IR and VCD,^4,18^ D_2_O solutions were used in these spectral techniques. The ROA spectra in D_2_O were measured as well and are plotted in Figure S1.

The Raman spectra below 400 cm^–1^ are rather monotonic, while for ROA, a large, more structured signal is present in this low-frequency region. The spectrometer allows measurements from approximately 50 cm^–1^. The disordered and α-helical L-PGA forms give predominantly positive ROA, but the detailed spectral patterns are different. The low-frequency pattern changes even more dramatically at pH 4, where more numerous and better resolved bands appear. This suggests a more compact crystal-like structure,^27^ compatible with the β_2_ β-sheet-based fibril models.^2,11^ Although low-frequency ROA is relatively unexplored, several studies have confirmed its usefulness and the unique information it provides on molecular structure and dynamics.^28–30^

In the fingerprint region (∼400–1800 cm^–1^), the disordered and α-helical ROA patterns are slightly similar. In the CH stretching region, only a small ROA band at 2952 cm^–1^ is visible for the disordered form. Therefore, IR and VCD appear to be more useful to distinguish between the two forms. In particular, VCD provides a typical w-shaped pattern for the amide I signal (around 1650 cm^–1^) in deuterated peptide and protein solutions.^4^ The fibrils provide the most distinct patterns in all four spectra types, such as the rich fingerprint shape in the Raman and ROA, measurable CH stretching signal, and split VCD and IR signals of amide I.

For the disordered structure, the fingerprint ROA spectrum is consistent with previous reports.^21,24^ The two strong positive ROA bands at 83 and 197 cm^–1^ for L-PGA resemble those reported for disordered α-synuclein, where they appeared at 97 and 185 cm^–1^, respectively.^20^ The strong positive band at 1320 cm^–1^ was assigned to amide III and ^α^CH bending modes.^19,21^

The IR and VCD spectra of the disordered conformation were also analyzed earlier;^5,6,10^ however, the weak positive amide II’ band at 1474 cm^–1^ and the negative CH bending band at 1369 cm^–1^ have not been reported so far. Despite the strong IR intensity of the COO^–^ antisymmetric stretch, no corresponding VCD signal is observed, likely due to the dynamic and isotropic orientation of the side chains in the disordered state.

Similarly, as for the disordered form, the ROA spectra of L- and D-PGA at pH 4.6 are reasonable ″mirror images″; some distortion is visible in the OH stretching region due to the high Raman scattering of water. Note that the α-helix of PGA could not be measured in the fully protonated form because it spontaneously aggregated when protonation was higher than ≈75%. Hence, the spectra may contain some contribution (∼25%) from disordered peptides. In the low-frequency ROA region, l-PGA exhibits two positive bands at 133 and 178 cm^–1^, which are at least twice as intense as the strongest midfrequency bands.

The mid (fingerprint) ROA spectra of the PGA α-helix were analyzed previously.^24^ The 133 cm^–1^ band (cf. Table S1 for the detailed frequency list) resembles the 128 cm^–1^ signal of α-helical poly-l-alanine^28^ and similar ones in other helical peptides,^19^ and indeed confirms the previous hypotheses that it may be used for helix detection. It is interesting that in D_2_O (Figure S1), a clear CH stretching couplet at 2920 (−) and 2960 (+) cm^–1^ is visible, undetectable in H_2_O. This suggests that a fine isotopic effect takes place, affecting either the stability of the α-helical geometry or the actual ROA signal. The isotopic and solvent effects are also known from IR and VCD measurements in organic solvents.^6,22^

To the best of our knowledge, previous reports of the ROA of PGA fibrils are not available. Apart from the low-frequency features (bands at 55, 74, 84, 110, 150, and 181 cm^–1^), the negative amide I band (1645 cm^–1^) is very interesting since the amide I vibration usually gives a positive or a split negative/positive signal in most peptides and proteins, including those with a high content of β-sheet.^19^ The amide I Raman signal is split, with a minor component at 1601 cm^–1^, which may indicate bifurcated hydrogen bonding.^3^ Similarly, as in VCD,^3,11,14^ the carboxyl C=O stretch gives an ROA couplet at 1727 (−) and 1738 (+) cm^–1^. The carboxyl C=O stretching frequency of 1731 cm^–1^ is higher than that of the α-helix (1712 cm^–1^), which may reflect changes in the hydrogen bonding as well as in its environment. In particular, the hydrophobic fibril core makes the C=O bond stronger and its stretching frequency higher.^31,32^

Finally, in the highest frequency regions, the fibrils gave a strong negative CH stretching band at 2930 cm^–1^ and a weaker positive one at 3000 cm^–1^. A similar shape was observed for the deuterated samples (Figure S1). The strength of the signal suggests that the side chains are fairly rigid and adopt a chiral conformation. The OH stretching region (∼3100–3700 cm^–1^) is peculiar, with an intense N–H stretching Raman band at 3344 cm^–1^, accompanied by a negative ROA signal at 3347 cm^–1^. It is not present in the other two PGA conformations or (as ND-stretching) in the spectra of the deuterated sample. Another “mystery” is the so far unobserved broad negative (∼3500 cm^–1^) ROA signal of the O–H stretching bands. This is obscured by the baseline distortion due to the huge water Raman signal in this region; nevertheless, a similar, albeit weaker signal is also visible in the deuterated spectra. The disordered and α-helical forms do not seem to have this feature. The signal thus may correspond to the net chiral orientation of the COOH (COOD) groups as well as the contribution of water molecules attached to the fibrils.

### Molecular Dynamics

Clearly, there are many challenges in the theory regarding a detailed understanding and interpretation of the spectra. Modeling can address at least some of the issues. MD simulations were based on free dynamics and semirigid models when the desired conformations were constrained. Figure 2 shows the resultant angular distributions of the backbone torsion angles (φ and ψ). Only the disordered structure modeled as PPII was reasonably stable under the Amber force field; therefore, restrained structures were used to model the α-helical, 3_10_, and β-sheet conformations. To mimic the fibrils, except for the one-strand models, a multistrand system was used, following previous modeling.^11^

**Figure 2:**
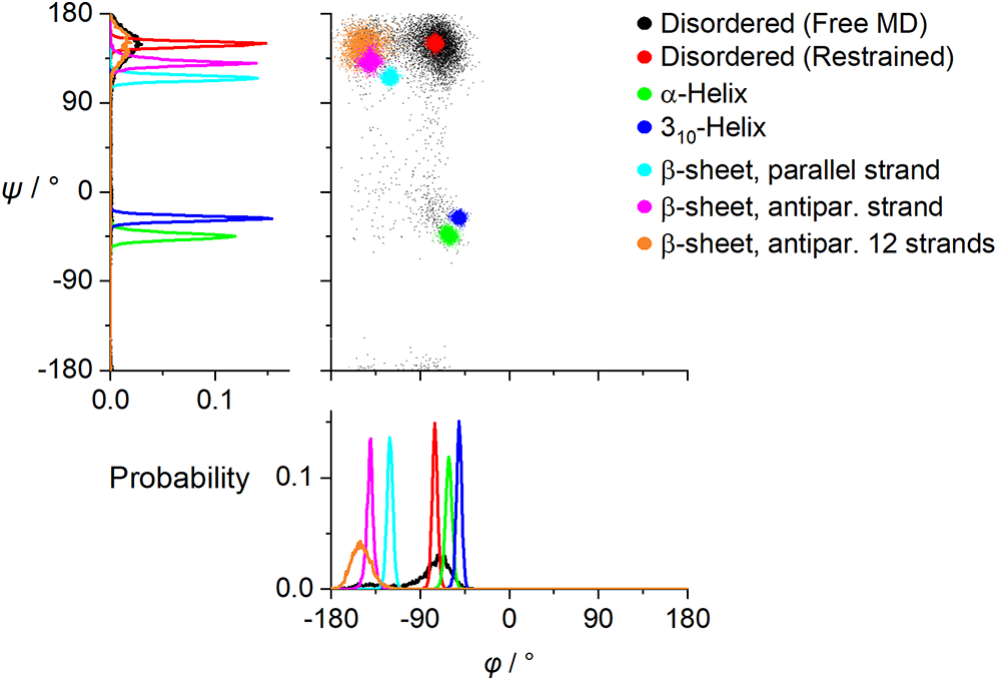
Distributions of the backbone torsion angles (φ, ψ) obtained by MD simulations for the disordered, α-helical, 3_10_-helical, and β-sheet L-PGA conformations.

### Implicit vs Explicit Solvent Models

To understand the role of the solvent in the spectral shapes, we simulated the spectra using the dielectric (implicit, CPCM) and combined CPCM with explicit solvent modeling. The simulated spectra are compared with the experimental results in Figure 3. One can see that although both approaches lead to reasonable agreement with the experiment, the explicit inclusion of a few hydrogen-bonded water molecules clearly provides results superior to those of the bare CPCM approach. Only for α-helical VCD is the overall agreement poor for both cases. Here, the experimental spectrum may be affected by the presence of other conformations, or the simulation may miss some finer isotopic effects.

**Figure 3:**
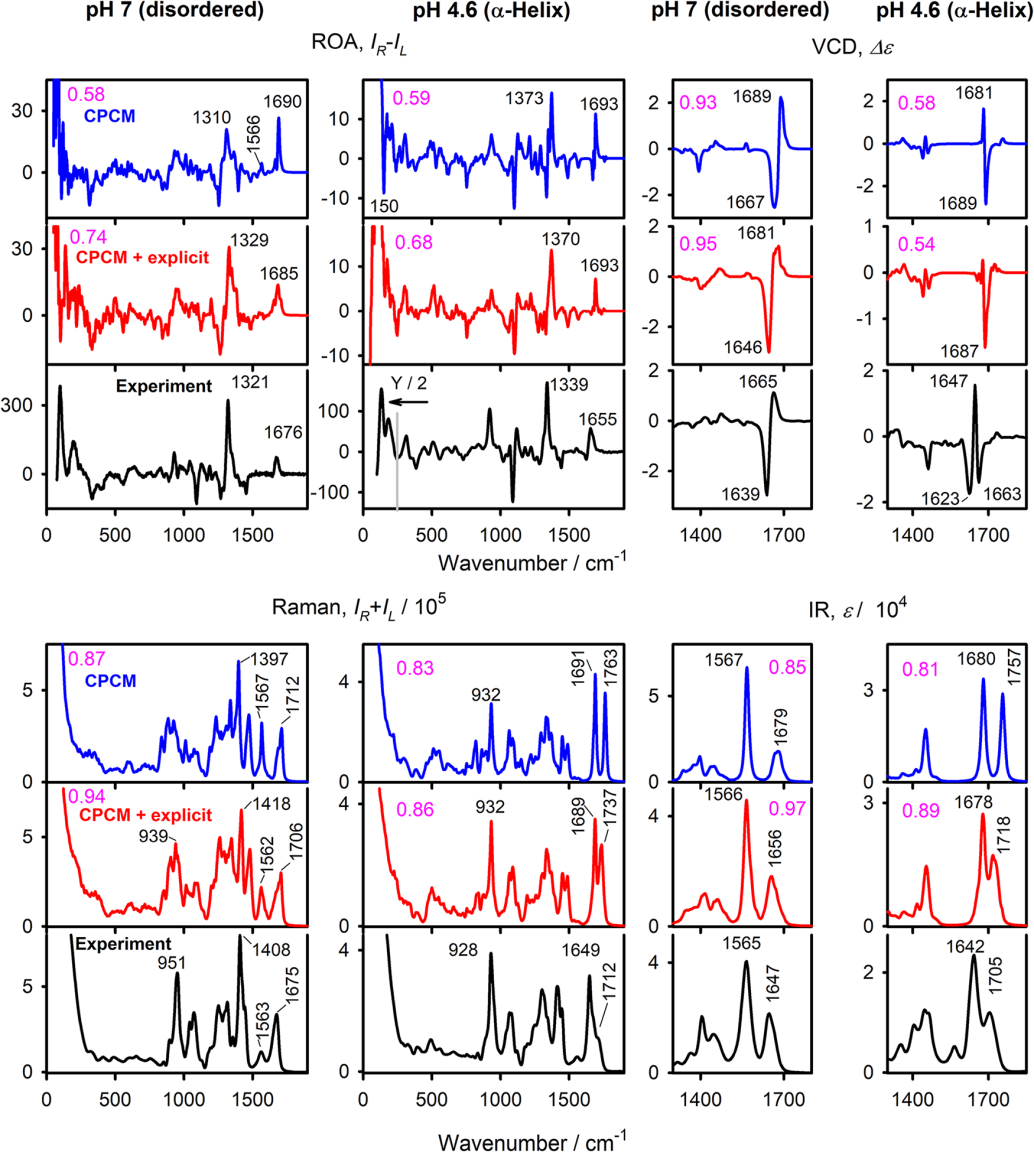
ROA and Raman (in H_2_O), VCD and IR (in D_2_O) spectra of disordered and α-helical L-PGA forms, calculation (B3LYP/6-311++G**) with implicit (CPCM, blue) and combined (red) solvent models, and experimental (black) results. Similarity factors for the experiment (magenta) and positions of the selected bands (black) are indicated. The experimental ROA of the α-helix in the low-frequency region was divided by two as indicated; the calculated frequencies were not scaled.

As expected, vibrational bands associated with polar groups capable of forming hydrogen bonds, such as backbone amides and side-chain carboxylates, benefit the most from explicit modeling. For example, the amide I ROA intensity is systematically overestimated by CPCM, for both the disordered and α-helical cases. At 150 cm^–1^, bare CPCM wrongly predicts a negative ROA for α-helix, which disappears after the explicit inclusion of water, which suggests an interplay between the vibrational motions of α-helices and their hydration. The carboxyl C=O stretching band is particularly affected for the α-helix, moving from 1763 cm in CPCM to 1737 cm^–1^ in the explicit model, closer to the experimental value (1712 cm^–1^). The amide I band is less affected by the intramolecular hydrogen bonding and does not change much with the explicit addition of water molecules.

We also tested the effect of flexibility on the spectra; i.e., for the disordered form, we generated spectra for the PPII-constrained and free MD (cf. Figure 2). It appears that the two spectral sets are quite similar; the ROA, Raman, IR, and VCD spectra are compared to the experimental results in Figure S2. IR and VCD are almost unaffected by the flexibility difference. For Raman, the restrained model reproduced the experimental shape better around 950 cm^–1^, while the free dynamics were statistically slightly better for ROA (similarities 0.72 vs 0.77). The spectra are, in principle, able to capture the differences in the geometry dispersion; however, at the present stage, this is challenging for the PGA due to experimental noise and the limited precision of the calculations.

Although experimental VCD was shown to be able to distinguish between α- and 3_10_-helices,^33–37^ this is not confirmed by our simulations for PGA, giving fairly similar spectra for both forms (Figure 4). This may be caused by the complicated C=O chromophore and specific behavior of PGA, compared to the usual 3_10_ models, which often contain nonstandard amino acids and organic solvents.^36^ Thus, the advantage of complementary measurement of ROA containing a larger wavenumber segment appears profitable since the ROA differences are more apparent, and the α-helical simulation clearly gives superior agreement with the experiment. For example, for the α-helix, the intensity ratio of the positive 1302 cm^–1^ and amide I band at 1649 cm^–1^ is reproduced at approximately 3:1, in agreement with the experiment, whereas the 3_10_-helix yields an almost 1:1 ratio. Similarly, the negative ROA peak at 1443 cm^–1^ (exp., assigned to CH_2_ scissoring) is more developed in the α-helical simulation. Below 200 cm^–1^, the α-helical model produces two intense overlapping peaks, consistent with the experiment, whereas the 3_10_-helix gives a much weaker signal.

**Figure 4:**
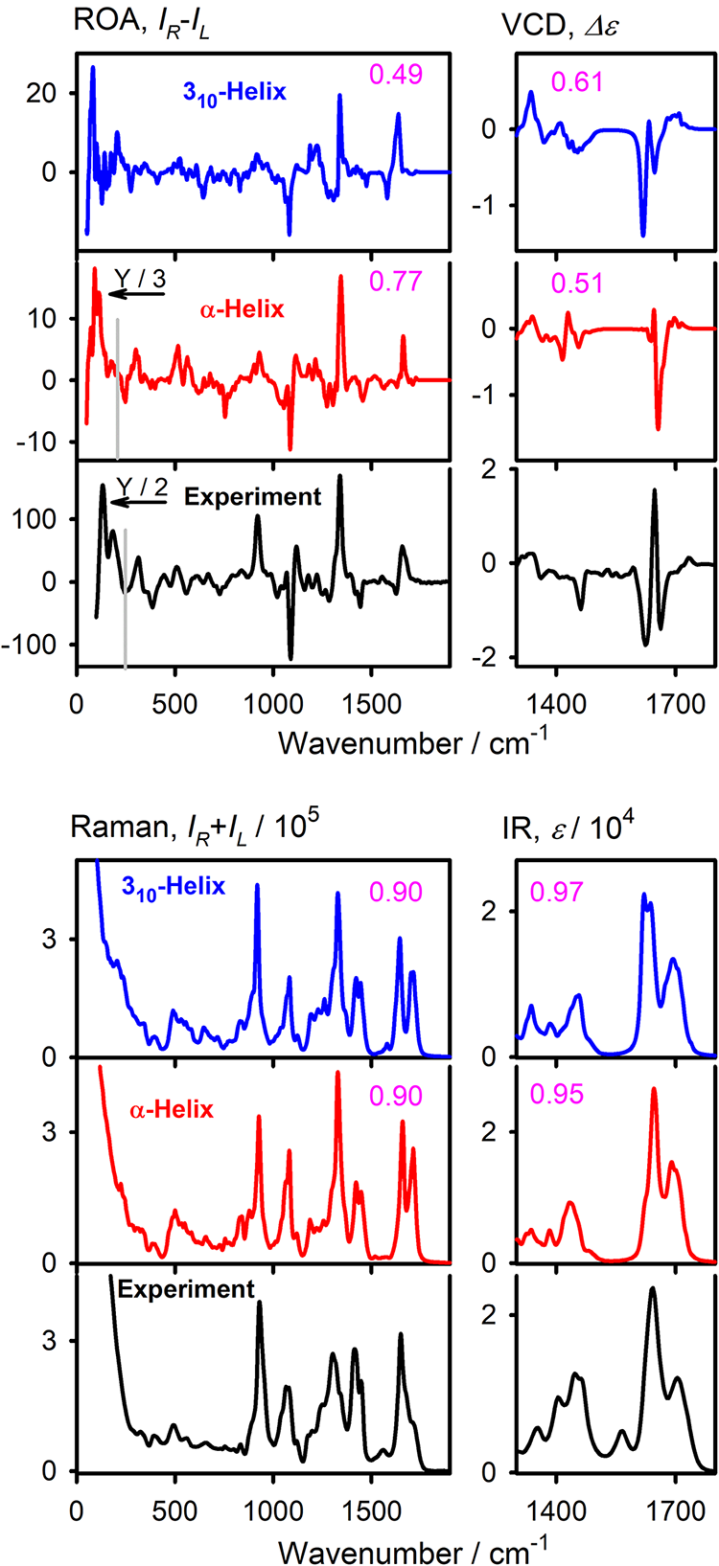
Calculated ROA, Raman, VCD, and IR spectra of α- and 3_10_-helical L-PGA vs experiment. ROA/Raman are for H_2_O, VCD/IR for D_2_O, CPCM + explicit solvation was used for the simulations, an average of 9 snapshots is shown, and calculated frequencies are scaled according to the experimental ones. Similarity factors for the experiment (magenta) and intensity scaling in some regions are indicated.

Finally, the computations and experiments are compared for the Raman and ROA spectra of the fibrils, as shown in Figure 5. For the Raman spectra, very realistic spectral shapes were obtained by the simulation, and the most important trends observed during the H_2_O → D_2_O exchange were reproduced. In the Raman spectra, the amide II band downshifts from 1560 to 1415 cm^–1^, where it overlaps with the CH bending signal. The amide III band at 1230 cm^–1^ shifts to 982 cm^–1^. In the ROA spectra, the amide II couplet at 1566 (−)/1553 (+) cm^–1^ [calc. 1523 (−)/1433 (+)] moves to 1484 (−)/1470 (+) cm^–1^ [calc. 1485 (−)/1402 (+)], and a new positive band appears near 1410 cm^–1^ (calc. 1329 cm). The strong positive amide III band at 1277 cm^–1^ (calc. 1255 cm^–1^) shifts to a much lower frequency and becomes a weak negative at 856 cm^–1^ (calc. 726 cm^–1^). A new negative band near 1021 cm^–1^ emerges upon deuteration in the experiment, whereas the calculation predicts only a much weaker feature at around 986 cm^–1^. The simulations also describe very well the deuteration effects for the disordered and α-helical forms (Figures S3 and S4). For example, deuteration causes an intensity decrease in the amide III region (around 1250 cm^–1^) related to the decoupling of N–H bending and C–N stretching vibrations.

**Figure 5:**
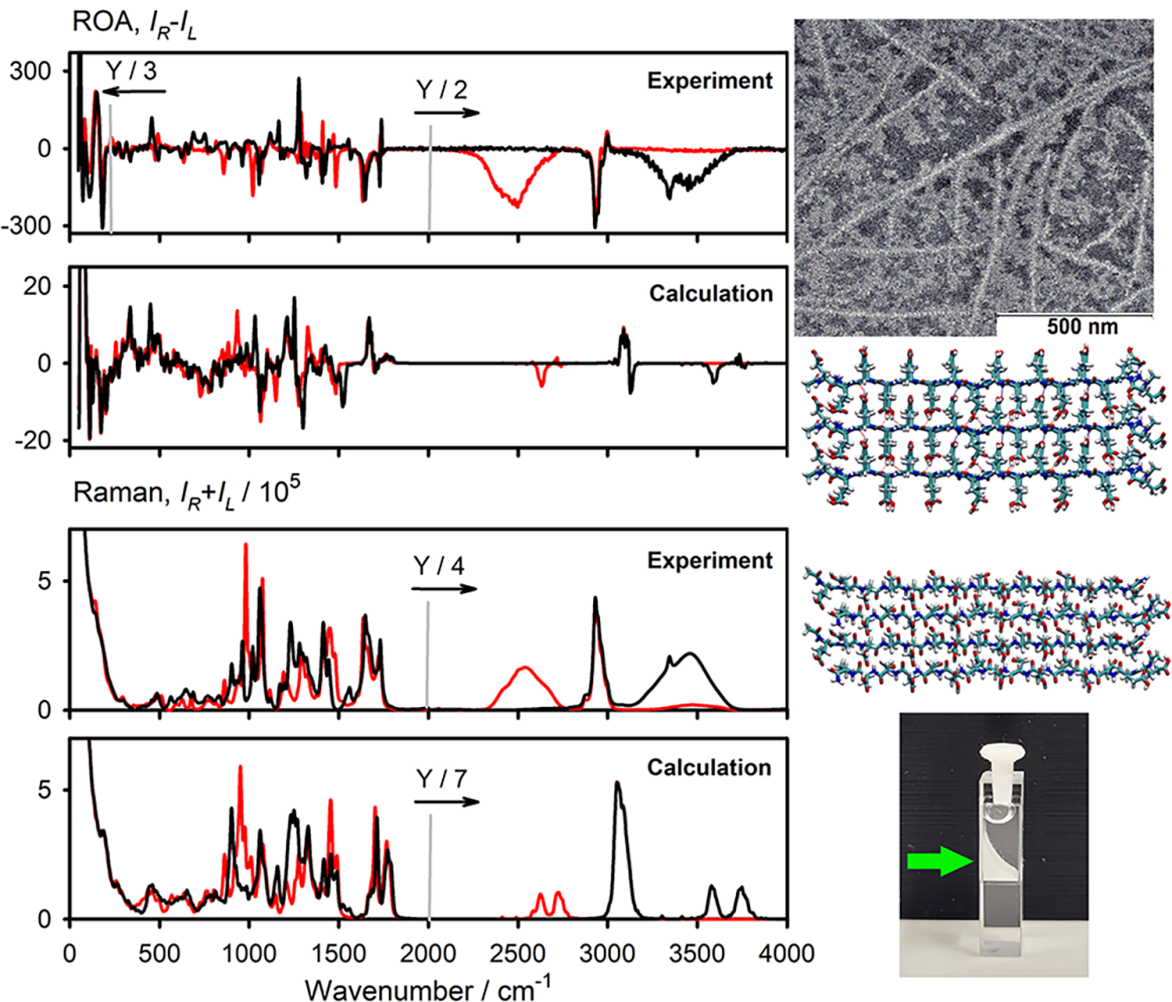
ROA and Raman spectra of L-PGA fibrils in H_2_O (black, experiment at pH 4) and D_2_O (red, pD = 4). On the right, two projections of the 12-chain model used in the simulation (1000 snapshot average) and a TEM image of the sample are shown. In the photo, the green arrow indicates the direction of the incident light. In the spectra, the intensities in some regions are divided as indicated.

For ROA, the quality of the simulation is much worse than that for the disordered and α-helical forms, which is attributed to the complexity of the system and uncertainty in the geometry. Although the β_2_ tightly packed model^2^ is generally accepted as the best guess of the fibril geometry,^38^ X-ray and detailed position of the glutamic side chains are not known; they are probably also not ideally reproduced by the MD force field. However, the compact multistrand model (Figure 5) gives more realistic spectra than the bare strands in β-sheet conformations (Figure S5). In addition, some interesting experimental trends were reproduced even for ROA, such as the negative signal of the NH stretching (exp. 3347 cm^–1^) and a stronger CH stretching signal (exp. within 2900–3000 cm^–1^). The computation still predicts an ND-stretching ROA for the deuterated system, where it is, however, not visible. This can be caused by limited instrumental sensitivity around 2500 cm^–1^, as this region lies at the edges of the two detectors used.^39^ The CH stretching signal is about the same in the deuterated and natural samples. The simulations do not reproduce this well, which can be partially attributed to the anharmonic effects not included in the computations.

A predominantly positive (more accurately, 1738 (+)/1727 (−) couplet) C=O stretching side-chain signal is predicted reasonably. A huge disappointment is the wrongly calculated sign of the amide I band, which is negative in the experiment, perhaps related to the fibril twist or intramolecular hydrogen binding pattern. The simulation is more reasonable in the CH bending and single-bond stretching (∼700–1500 cm^–1^) region, where most of the strongest bands are reproduced with the correct signs. Below 200 cm^–1^, the computed strong, sharp bands are in qualitative agreement with the observation, although the positive signal at 150 cm^–1^ is predicted with a much lower intensity.

## Conclusion

We measured the VCD, IR, Raman, and ROA spectra of PGA enantiomers in the natural and deuterated states. The measurements with both enantiomers led to mirror-image spectra, thus confirming the experimental intensities. This appeared particularly critical for ROA measurements of PGA fibrils, which were often hampered by artifacts. Multiscale molecular dynamics and density functional theory simulations provide an excellent basis to assign the observed spectral bands and to relate the experimental shapes to the structure. In the future, however, better models of the fibril geometry are needed. We view the extension of this spectroscopy to the peptide and protein aggregates, together with theoretical improvements, as important for many applications connected to biological research. For PGA, the experimental spectra could be rationalized to a great extent and connected to the structure through multiscale molecular dynamics and density functional theory computations.

## Experimental Methods

### Sample Preparation

Sodium salts of poly-l-glutamic acid (L-PGA, cat. no. P4761) and poly-d-glutamic acid (D-PGA, cat. no. P4033) were purchased from Sigma-Aldrich. Using mass spectrometry, an average molecular weight of approximately 3 kDa was determined, with approximately the same distributions for L- and D-enantiomers (Figure S6). Stock solutions of PGA were prepared at a concentration of 100 mg/mL in Milli-Q H_2_O and D_2_O. For the latter case, the initial stock solution was lyophilized and redissolved in D_2_O to ensure complete H/D exchange. The pH was monitored by indicator strips (Merck). For the disordered form, the pH/pD was adjusted to 7 with 1 M NaOH. To prepare α-helix, pH/pD were lowered to 4.6 with 0.25 M HCl/DCl. Based on the ratio of the amide and carboxyl C=O stretching bands, the α-helix/disordered ratio was 75/25 in such samples; it was impossible to synthesize pure α-helix, as it would aggregate to β-sheet fibrils. For the fibrils, the pH/pD was adjusted to 4.0 with 0.25 M HCl/DCl, and fibril formation was allowed to proceed for approximately 1 h at room temperature, which was monitored by the increase in sample turbidity.

### Spectra Measurement

The IR and VCD spectra of the deuterated samples were recorded on a ChiralIR-2X spectrometer (BioTools Inc., Jupiter, FL) at a resolution of 8 cm^–1^. The samples were placed between two CaCl_2_ windows separated by a 55 μm spacer, requiring a sample volume of approximately 30 μL. The final concentration of PGA in all measurements was 23 mg/mL. For the disordered and α-helical states, the spectra were accumulated for approximately 10 h. For the β-sheet fibrils, the pH was adjusted to 4.0, and the sample was incubated for 1 h at room temperature before data collection. The spectra were then accumulated continuously for 10 h. The reported spectrum is an average over the final 2 h, by which time the spectral features stabilized. The background spectrum of pure D_2_O was subtracted from both the IR and VCD spectra.

The Raman and Raman optical activity (ROA) spectra were measured on a spectrometer at the Palacký University, Olomouc,^39^ using 532 nm laser excitation. For the disordered and α-helical states, the samples were prepared as for VCD. The fibrils were incubated at 60 °C for 3 days, and the suspension was transferred to a fused silica cell (volume 80 μL) and centrifuged at 1000*g* for 30 s so that the fibrils were concentrated at the bottom and along one side of the cuvette, where the laser beam was focused (Figure 5). The laser power at the sample was 240–400 mW, and the acquisition times were 6–30 h (Table S2). The glass and solvent (H_2_O or D_2_O) spectra were subtracted from the Raman spectra. Apart from the standard scattered circular polarization (SCP), dual circular polarization modulations (DCPI, DCPII) were used to rule out significant birefringence in the fibril samples (Figure S7).

The secondary structural changes are also indicated by electronic circular dichroism spectra and their standard analysis (Figure S8).

### Molecular Dynamics (MD)

MD simulations were performed using the Amber suite of programs.^40^ Initial backbone dihedral angles (φ, ψ) were set to canonical values^41^ (in deg): disordered = polyproline II helix (−75, 150), α-helix (−60, −45), 3_10_-helix (−49, −26), parallel (−140, 130), and antiparallel (−120, 115) β-sheet. The single-strain models contained 30 glutamic acid residues. A multistrand fibril model based on a previous modeling^11^ contained three stacked four-stranded antiparallel β-sheets. For all conformations, except PPII, the side chains were protonated. The peptide was placed in a rectangular periodic box filled with water and sodium counterions (for the PPII). Harmonic restrain potential was applied for the backbone angles (r1, r4 = ±5°; rk2,3 = 128 kcal/mol), the systems were minimized, and subjected to 1 ns MD equilibration and 10 ns production run, with 1 fs integration time, NVT ensemble, 300 K temperature, ff14SB^42^ (peptide) and TIP3P^43^ (water) force fields. For PPII, a free MD was run as well.

### Spectra Simulation

For selected snapshots, all or distant (>3 Å from the peptide) and non-hydrogen-bonded water were deleted, and the CCT method was used to generate the spectra.^16,17,44^ For this, smaller overlapping fragments containing 4 (PPII) and 6 (helices) amino acid residues were prepared, where the vibrational parameters could be calculated. For the fibrils, smaller fragments containing 4 amide bonds and larger ones, including cross-sheet interactions (Figure S9), were prepared. The fragments were then partially geometry optimized in normal mode coordinates,^31,45,46^ constraining normal modes of frequencies below 100 cm^–1^, and the harmonic force field and intensity tensors were calculated.^47^ The Gaussian program^48^ was used for the quantum chemistry calculations, with the B3LYP^49^ functional and 6-31++G** or 6-31G** (smaller and bigger fragments) basis sets. The aqueous environment was simulated using the CPCM^50–52^ solvent model. The force field and tensors were transferred back to the whole peptide, and vibrational frequencies and spectral intensities^53^ were calculated. Smooth spectra were generated using Lorentzian profiles of 10 cm^–1^ with a full width at half-maximum. Simulated Raman and ROA frequencies for Figures 4 and S2 were scaled with variable factors to simplify comparison with experiment (Table S3). Comparing the calculated spectra *S*_cal_ with the experimental spectra *S*_exp_, similarity factors were computed as

$$s=\frac{\int S_{\mathrm{cal}}\left(\omega \right)S_{\exp}\left(\omega \right)d\omega }{\sqrt{\int S_{\mathrm{cal}}^{2}\left(\omega \right)d\omega \int S_{\exp}^{2}\left(\omega \right)d\omega }}$$

The integration ranges were 230–1800 cm^–1^ for Raman/ROA, and 1250–1800 cm^–1^ for IR/VCD, and the calculated spectra were scaled before comparison.

## Supplementary Material



## Data Availability

The data that support the findings of this study are openly available in Zenodo at https://zenodo.org/records/17515528.

## Notes

Additional figures, band assignment tables, ECD, and MALDI spectra. (PDF)
