# Supplementary material for: Three Conformations of Polyglutamic Acid Monitored by Vibrational Optical Activity
Source: Anal Chem. 2025 Dec 12;97(50):27913–20. doi: 10.1021/acs.analchem.5c05531 (PMC12750409; doi:10.1021/acs.analchem.5c05531)
Supplement: Supplementary file 1 [file ac5c05531_si_001.pdf]

Supporting Information

Three Conformations of Polyglutamic Acid Monitored by Vibrational Optical Activity

Andrii S. Kurochka,*[a] Jana Hudecová,*[b] Josef Kapitán,[b] Jiří Kessler,[a] and Petr Bouř[a]

[a] Institute of Organic Chemistry and Biochemistry, Academy of Sciences, Flemingovo náměstí 2, 16610 Prague, Czech Republic

[b] Department of Optics, Palacký University Olomouc, 17. listopadu 12, 77900 Olomouc, Czech Republic

* andrii.kurochka@uochb.cas.cz, hudecova@optics.upol.cz

Contents

Figure S1. Experimental ROA (I_R - I_L) and Raman (I_R + I_L) spectra of L (black) and D (red) PGA in D_2O, at concentration 100 mg/ml, for three pD values.

Figure S2. ROA, Raman spectra in H_2O and VCD, IR spectra in D_2O of L-PGA in the disordered state, simulations with free and restrained MD.

Figure S3. Raman and ROA spectra of disordered L-PGA in H_2O and D_2O.

Figure S4. Raman and ROA spectra of α-helical L-PGA in H_2O and D_2O.

Figure S5. Calculated Raman and ROA spectra of L-PGA single-strand β-sheet.

Figure S6. Molecular weight distribution of L- and D-PGA.

Figure S7. SCP, DCPI and DCPII Raman and ROA spectra of L-PGA in fibrillar form.

Figure S8. Experimental ECD spectra.

Figure S9. A fragment containing non-covalent interactions in the fibrils.

Table S1. Experimental frequencies of selected bands, assignment based on the computations.

Table S2. ROA acquisition times.

Table S3. Experimental and calculated frequencies used in the frequency scaling.

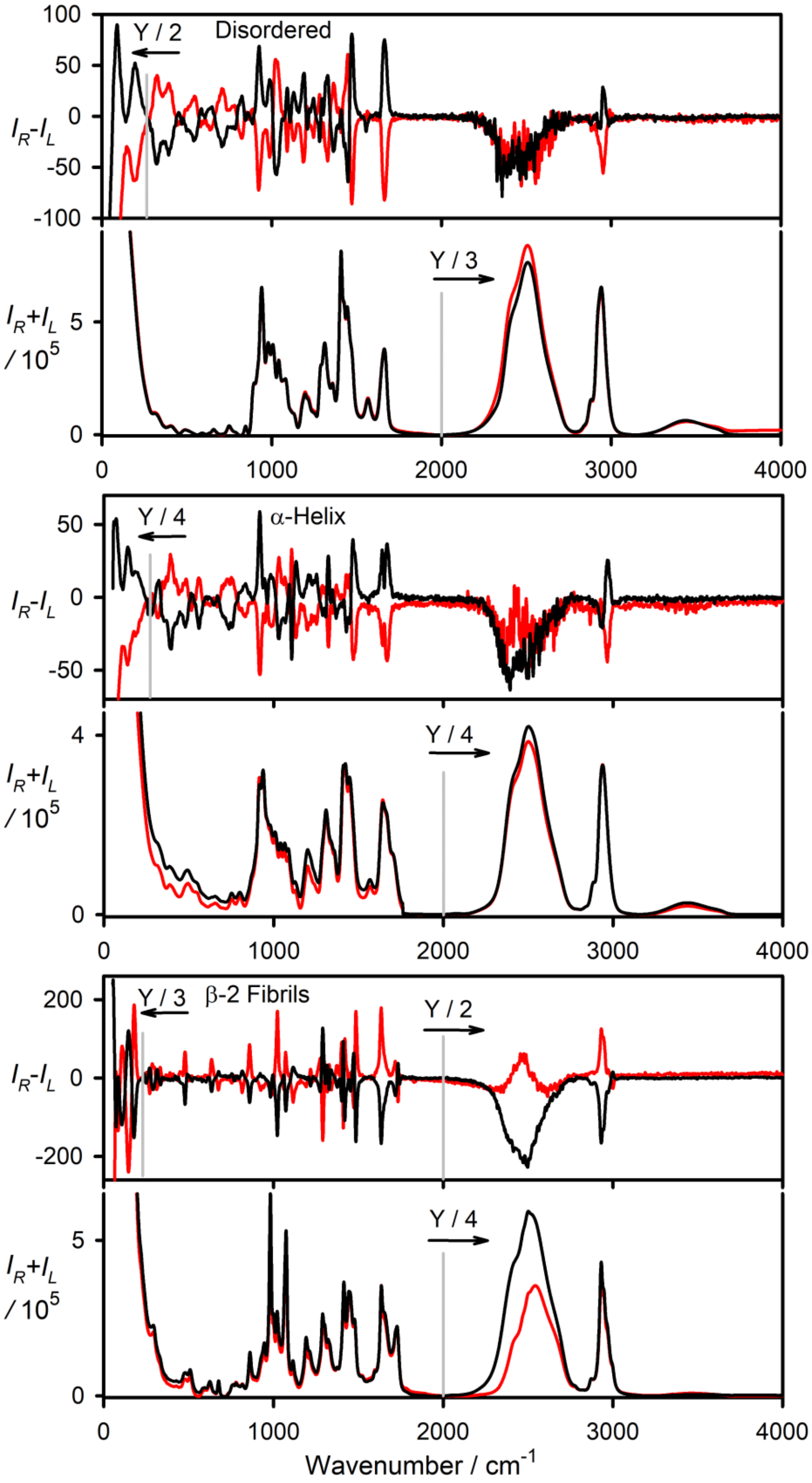

Figure S1. Experimental ROA (I_R - I_L) and Raman (I_R + I_L) spectra of L (black) and D (red) PGA in D_2O, at concentration 100 mg/ml, for three conformations. Intensities in some regions were divided as indicated.

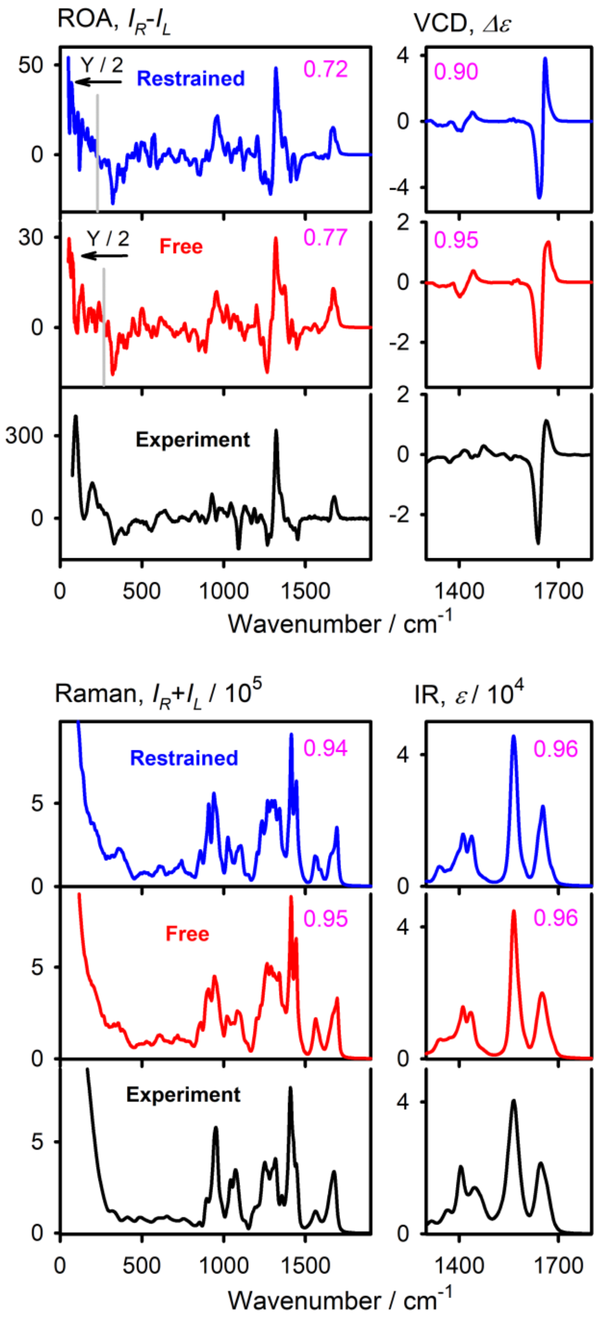

Figure S2. ROA, Raman spectra in H_2O and VCD, IR spectra in D_2O of L-PGA in the disordered state, simulations with free and restrained MD, and the experiment. For the simulations, the explicit solvation was used, average of 14 snapshots. Variable scaling of the calculated wavenumbers was used to enhance the comparison to experiment. Intensities in some regions were divided as indicated. Similarity factors to experiment (magenta) are indicated.

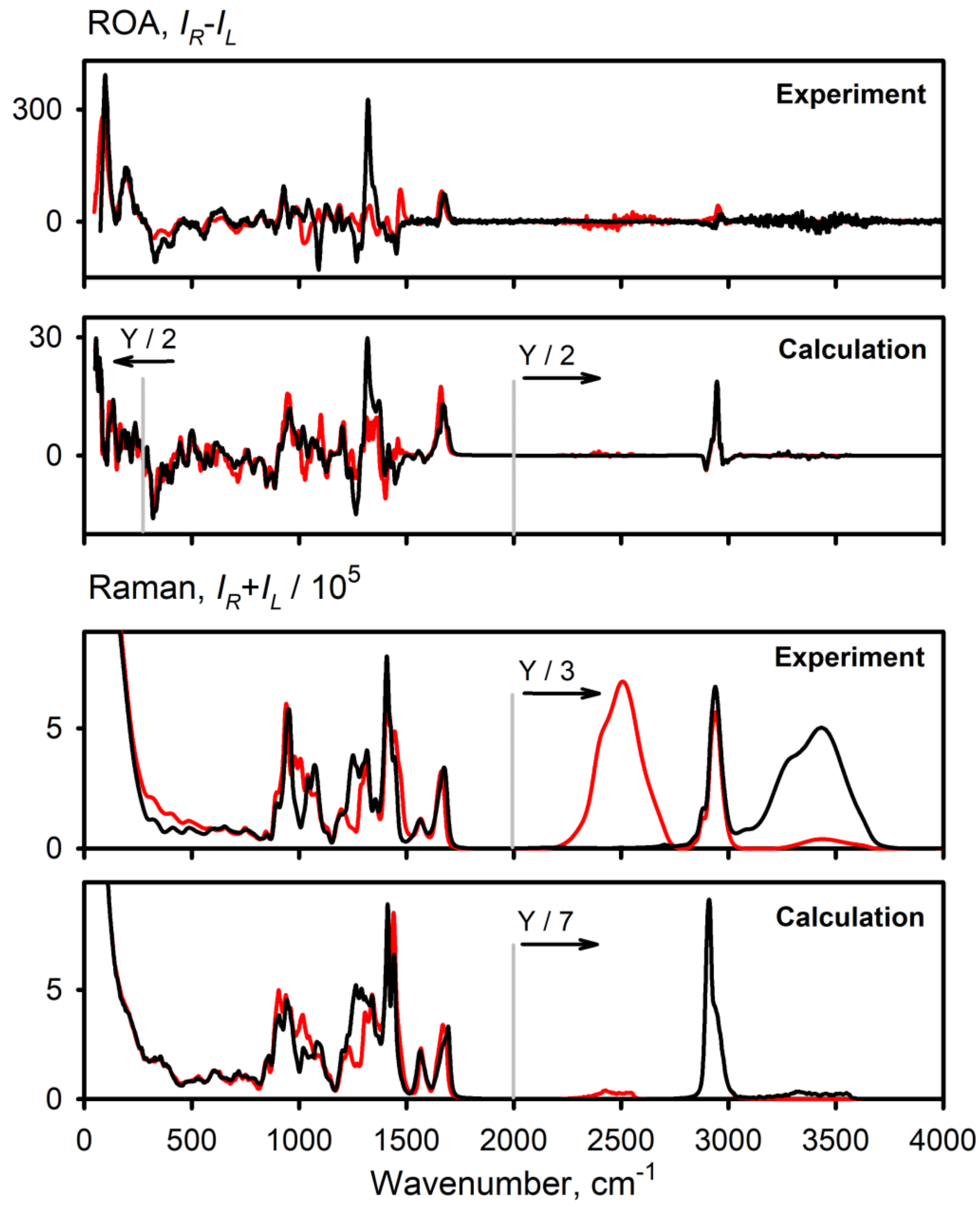

Figure S3. Raman and ROA spectra of L-PGA in the disordered conformation in H_2O (black) and D_2O (red), experiment (pH 7.0, 100 mg/ml) and simulation (14 snapshot average). Intensities in some regions were divided as indicated.

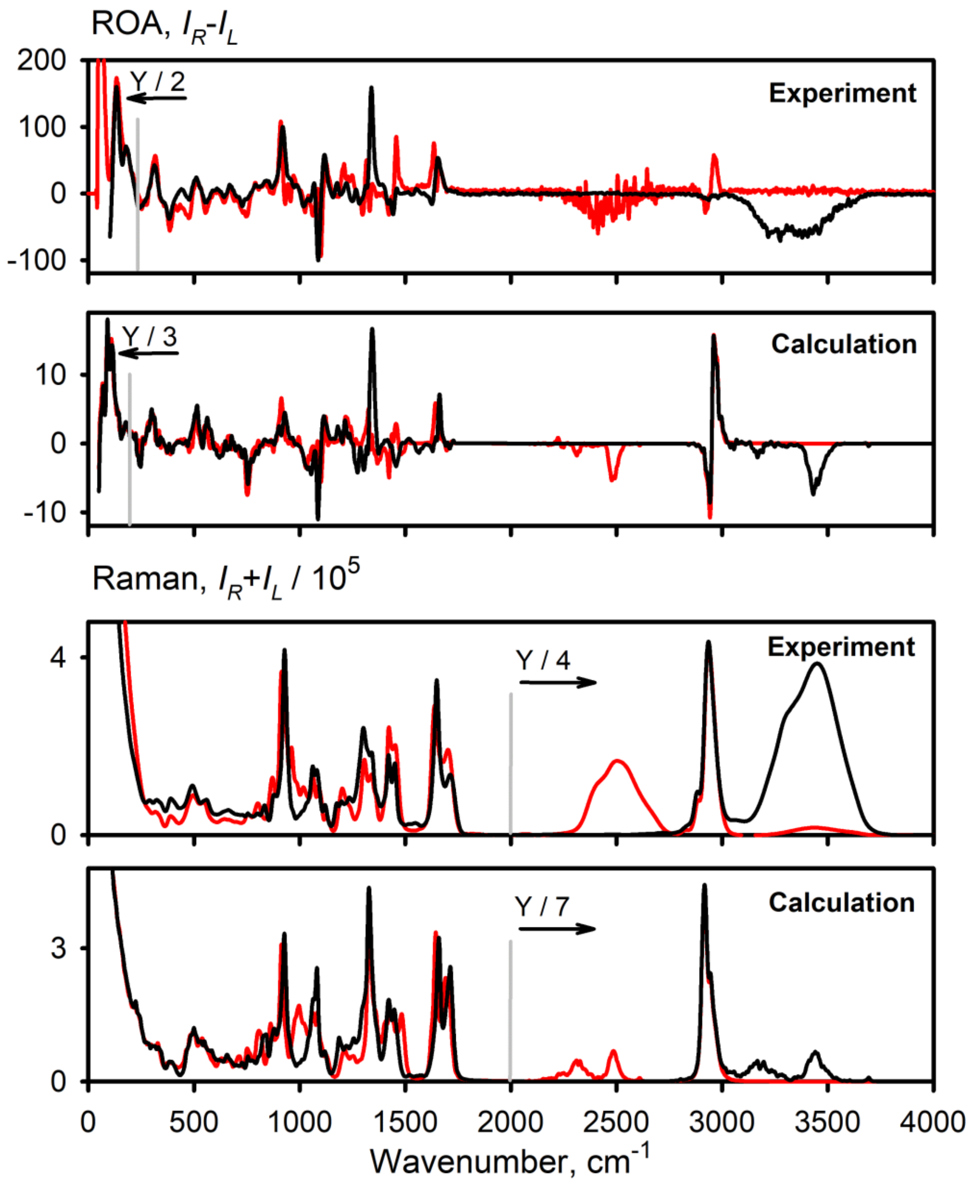

Figure S4. Raman and ROA spectra of L-PGA in the α-helical conformation in H_2O (black) and D_2O, experiment (pH 4.6, 100 mg/ml) and simulation (9 snapshot average). Intensities in some regions were divided as indicated.

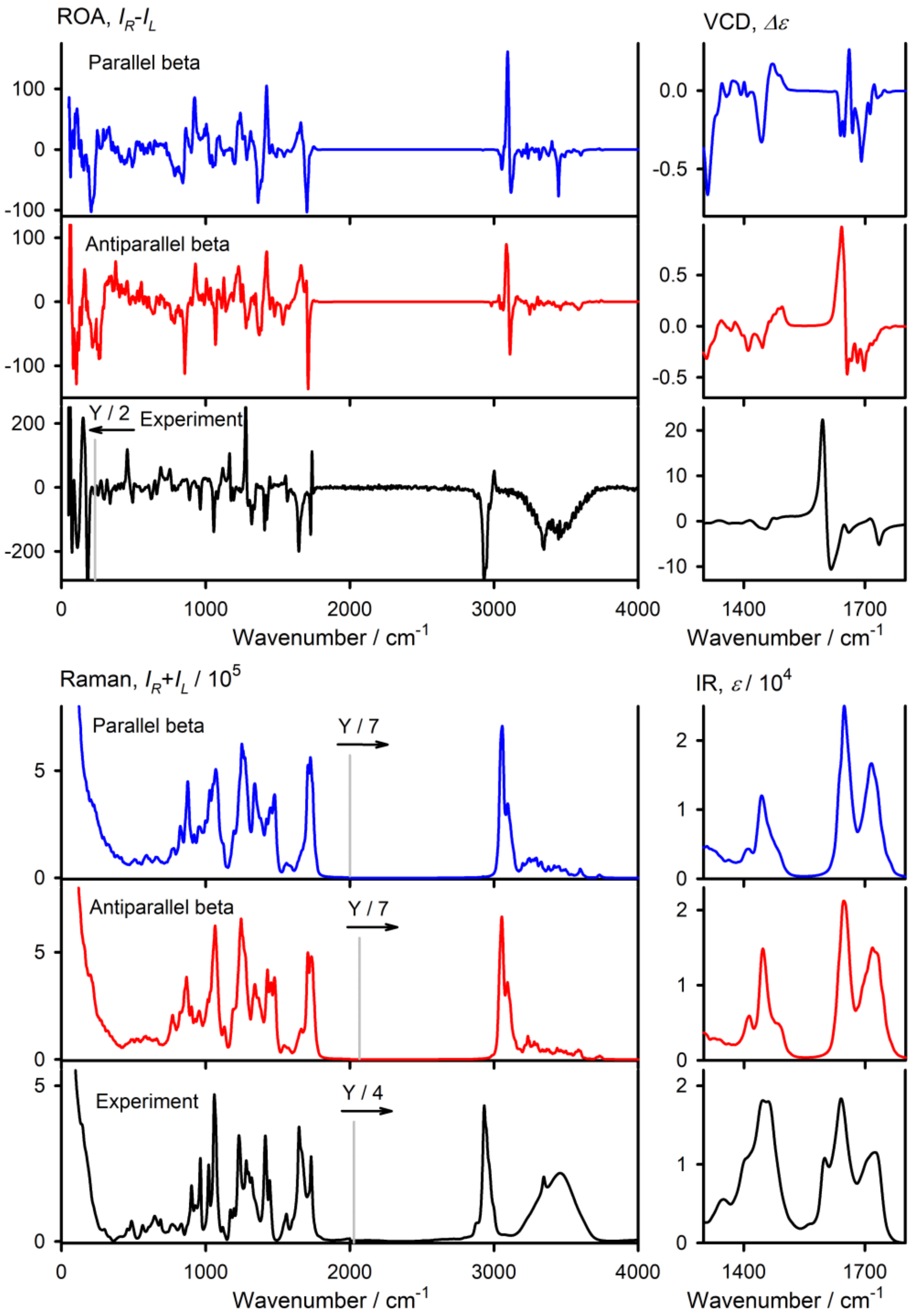

Figure S5. Left: Calculated ROA and Raman spectra of L-PGA single-strand β-sheet vs. experiment in H_2O. Right: Calculated VCD and IR spectra of L-PGA single-strand β-sheet vs. experiment in D_2O. The CPCM+explicit solvation was used for the simulations, average of 9 snapshots is shown, simulated frequencies are not scaled. Intensities in some regions were divided as indicated.

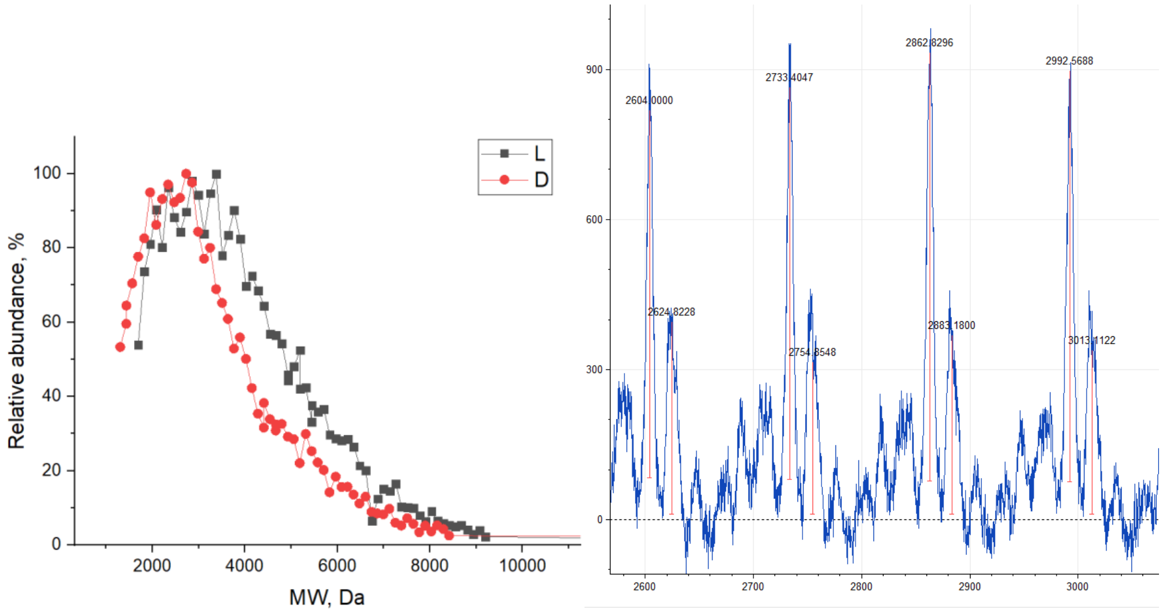

Figure S6. Molecular weight distribution of *L*- and *D*-PGA obtained by mass-spectroscopy using UltrafleXtreme MALDI-TOF/TOF (Bruker) in negative mode at pH 7. The biggest peaks are evenly distributed with average mass difference 129.3 which corresponds to one residue of glutamic acid, or 129.3+22.0 for sodium salt.

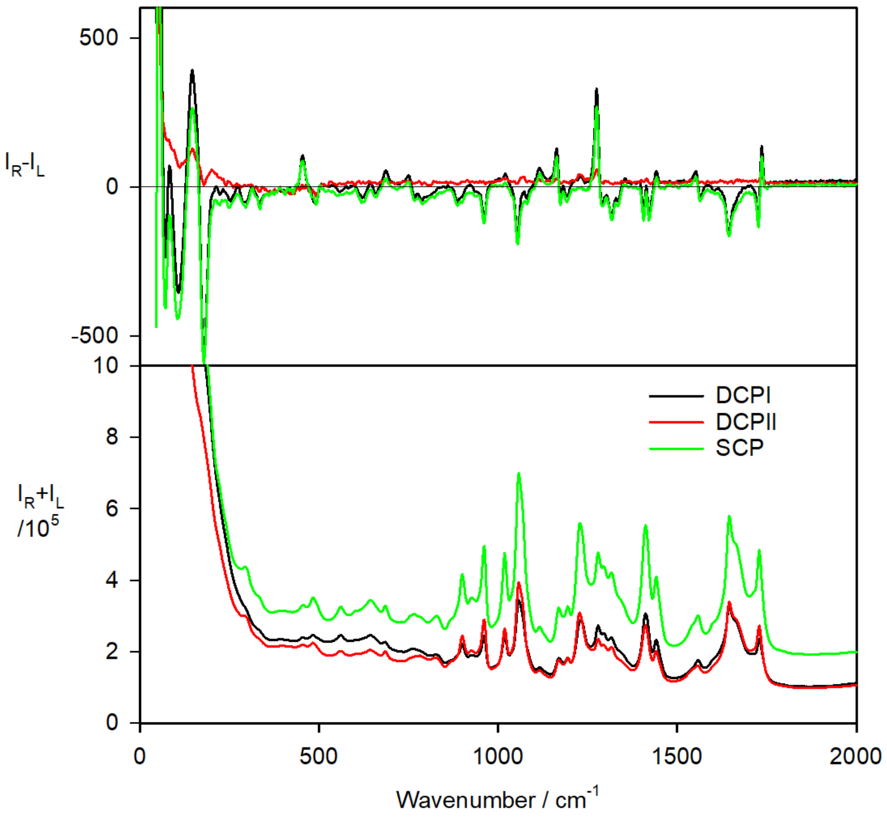

Figure S7. SCP, DCPI and DCPII Raman and ROA spectra of L-PGA in fibrillar form. DCPI and DCPII polarized Raman spectra can provide information of sample birefringence since strongly birefringent sample usually depolarizes DCP Raman spectra. Since observed SCP ROA is similar to DCPI ROA and DCPII spectra are close to zero, strong birefringence can be excluded in this case.

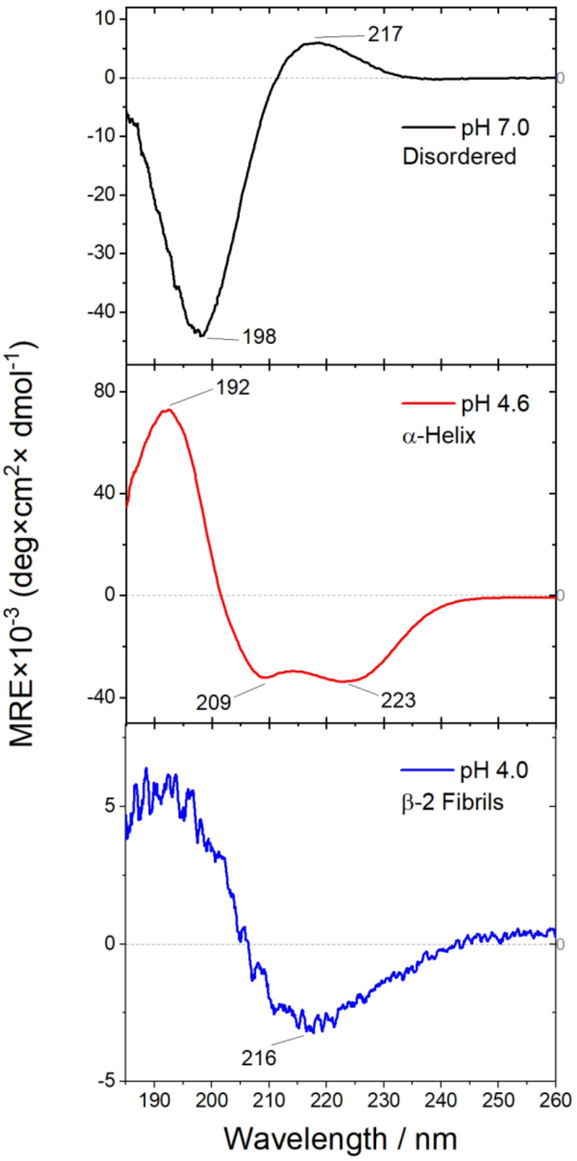

pH	α-Helix	β-Sheet	Turns	Disordered
7.0	3.10	6.50	23.40	68.20
4.6	76.50	0.40	8.90	17.50
4.0	0.00	46.60	19.60	33.40

Figure S8. Experimental ECD spectra of PGA, measured at pH 7, 4.6 and 4.0, and percentages of secondary structures as determined by the Chirakit software.

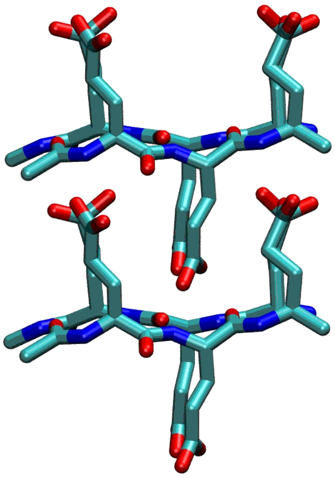

Figure S9. A fragment [Ac-Glu2-CONHCH-(CH2CH2CO2H)-Me • NH2-Glu3-COMe]$_2$ containing non-covalent interactions between the β-sheets, used to generate the spectra of the fibrils.

Table S1. Experimental frequencies of selected bands, assignment based on the computations

Abbreviations: vs = very strong, s = strong, m =medium, w = weak, vw = very weak, sh = shoulder.

1) Raman / ROA spectra in H_2O

	Disordered		α-Helix		β-sheet	
	Raman	ROA	Raman	ROA	Raman	ROA
v(N-H)	-	-	-	-	s 3344	m -3347
v(C-H)	vs 2939, sh 2883	vw +2952	vs 2936, sh 2884	vw +2937	vs 2931, sh 2874	w +3000 s -2930
v(C=O), COOH	-	-	m 1712	-	s 1731	m +1738, m -1727
v(C=O), Amide I	s 1675	m +1675	s 1649	m +1654, w - 1625	sh 1667, s 1647, vw 1601	s -1645
v(C=O), CO_2^- antisym.	m 1563	-	-	-	-	-
Amide II	m 1563	-	vw 1548	vw +1550	w1560	w-1566, w+1553
δ(O-H), CH_2 scissoring		-	m 1450	vw +1460, m -1443	m 1443,	m+1445
CH_2 scissoring	sh 1443	m -1450,	-	-	-	-
v(C=O), CO_2^- sym.	vs 1408	-	-	-	-	-
δ(O-H), CH_2 scissoring	-	-	m 1421	w -1419	s 1413	w -1423, w -1409
δ(C-H)	w 1355	sh +1344	sh 1341,	vs +1339	sh 1347	sh -1339
Amide III (δ(N-H) + *v*(C-N)), δ(αC-H)	m 1316, m 1251, b 1193	vs +1320, m -1287, m -1265, w -1202, w +1188, w -1169	s 1302, w 1235, w 1198, w 1176,	w -1282, w +1267, w -1249, w +1221, w -1191, w +1177, w -1159	m 1319, m 1282, s 1230, w 1196, w 1171,	m-1319, w-1294, vs +1277, w -1194, w -1176, m +1165
v (C-N), *v*(C-C), δ(C-H)	m 1072, w 1042	s -1089, w +1044, w -1024	w 1120, m 1081, m 1062	m +1118, s -1089, w +1068, w - 1018	vw 1118, vs 1060, s 1021,	w +1119, w – 1083, s -1057,
oop COOH	-	-	s 929,	s +920	s 964, m 902	m -961, w -888
v(C-O), *v*(C-C), CH_2 rocking	vs 952,	w -952, m +927	s 929,	s +920	s 964, m 902	m -961, w -888
CH_2 rocking, wagging, *v*(C-C)	sh 896, vw 852, vw 754	w -889, w -850, w -784	sh 880, w 834	w -869, w -728,	w 833, w 768, w 732,	vw -767, w +751
N-H oop bending, delocalized collective modes, backbone deformation	vw 652, vw 607, vw 488, vw 412, vw 316	m +639, m -556, m -394, s -330, s +197, s -140, vs +83	vw 559, w 493, vw 390, vw 324, vw 290	w -552, w +511, w -475, m -381, m +313, m -237, m +178, w – 160, vs +133	w 687, w 646, w 563, w 485, w 457, sh 334, w 298, m 144	w +688, w -661, vw +647, w -620, w -493, m +456, vs -181, s +150, m -110, w +94, m-74, vs +55

2) IR / VCD spectra in D_2O

	Disordered		α-Helix		β-sheet	
	IR	VCD	IR	VCD	IR	VCD
v(C=O), COOD	-	-	m 1704	w +1733	m 1729	m -1736,<br>w +1713
v(C=O), Amide I	s 1647	m +1664,<br>s -1639	s 1642	m -1663,<br>s +1648,<br>s -1624,<br>w +1589	m 1640,<br>m 1600	w -1659,<br>w +1643,<br>s – 1616,<br>vs +1595
v(C=O), CO_2^- antisym.	vs 1565	-	-	-	-	-
Amide II, CH_2 scissoring	m 1447	w + 1474	sh 1461,<br>m 1447	m -1462	sh 1460,<br>m 1447	w +1472,<br>m -1450,
v(C=O), CO_2^- sym.	s 1405	-	-	-	-	-
δ(C-H)	w 1366,<br>w 1317	w - 1369	m 1405,<br>w 1353	-	sh 1402,<br>w 1343	w +1414,<br>w +1337

3) Raman / ROA spectra in D_2O

	Disordered		α-Helix		β-sheet	
	Raman	ROA	Raman	ROA	Raman	ROA
v(C-H)	vs 2941,<br>sh 2883	vw -2987,<br>m +2951,<br>vw -2871	vs 2936,<br>sh 2887	s +2960,<br>m -2920	vs 2932,<br>sh 2874	m +2997<br>s -2931
v(C=O), COOD	-	-	s 1702	-	m 1728	vw -1743,<br>m +1732,<br>w -1714
v(C=O), Amide I	s 1663	s +1664	s 1639	m +1663,<br>w -1647<br>s +1635	sh 1657,<br>s 1637,<br>vw 1599	vs -1634
v(C=O), CO_2^- antisym.	m 1567	vw -1562	-	-	-	-
CH_2 scissoring	sh 1472	s +1472,	m 1452	s +1457	sh 1477,<br>s 1450	s -1484,<br>m +1470<br>w +1443
Amide II, CH_2 scissoring	w 1445,	s -1450,	s 1423	m -1423	s 1415	m -1418,<br>s +1410,<br>m -1400
v(C=O), CO_2^- sym.	sh 1424,<br>vs 1409	vw -1428,<br>w +1409				
δ(C-H)	w 1357,<br>s 1312,<br>sh 1290,<br>vw 1250,<br>sh 1213,<br>m 1196	m -1363,<br>m +1329,<br>w +1295,<br>m -1282,<br>w +1246,<br>m -1207,<br>m +1189	m 1340,<br>m 1306,	w +1346,<br>m – 1332,<br>m +1316,<br>m -1297,	sh 1322,<br>m 1291,	m +1339,<br>m -1327,<br>m +1316,<br>m -1304,<br>s +1289,<br>m -1272,
CH_2 twisting, δ(C-H)			m 1202	w +1249,<br>m +1210	m 1193	w -1217
v (C-N), *v*(C-C), δ(C-H)	sh 1125,<br>w 1079,	m +1129,<br>m +1091,	w 1125,<br>m 1089,<br>m 1064,	m +1126,<br>s -1100,<br>m -1063,	w 1115,<br>vs 1074,	m +1114,<br>s -1071,
Amide III (δ(N-H) + *v*(C-N)), δ(αC-H), *v* (C-N), *v*(C-C),	m 1041,<br>s 1006,<br>s 979,	s -1023,<br>m +987,<br>s +924	m 1018,<br>m 961,	m -1026,<br>m +971,<br>w -957,	m 1022,<br>vs 982,<br>w 945,	vs -1021,<br>w +995,<br>s -856,

	vs 940			w -933		
CH_2 rocking, wagging, *ν*(C-C)	s 898,<br>w 845,<br>w 749,	w -886,<br>vw -845,<br>m +823,<br>w -782,	s 914,<br>m 871,<br>w 802,<br>vw 755	s +911,<br>w +840,<br>w+790,<br>m -750	w 862,	w +815
CO_2^- and NHCO oop deformation, CH bending, backbone deformation	w 657,<br>w 589,	m -705,<br>vw +638,<br>vw +581	vw 645	w 642,	vw 676,<br>vw 626	w +675,<br>m -635
Oop N-H bending, O-D bending, CH_2 rocking, C=O bending of amide		w -545,	w 546,<br>w 496	w -553,<br>w +509,<br>w -476	w 509,<br>w 480	m +491,<br>m -476
Delocalized collective modes	w 490,<br>w 404,<br>m 316	m -391,<br>m -324,<br>s +194,<br>m -144,<br>vs 85		m -385,<br>m +316,<br>w -276,<br>w -249,<br>s +177,<br>vs +135,<br>vs >50	m 291	m -330,<br>m -286,<br>vs -176,<br>vs +144,<br>vs -107,

Table S2. Acquisition times used in ROA measurements

Solvent	Conformation	Enantiomer	Duration, h
H_2O	Disordered	L	26
		D	28
	α-Helix	L	36
		D	2
	β-sheet	L	7
		D	7
D_2O	Disordered	L	33
		D	36
	α-Helix	L	31
		D	28
	β-sheet	L	26
		D	26

Table S3. **Experimental and calculated frequencies (cm^{-1}) as used for the interpolative scaling of calculated wavenumbers.**

Disordered		Helix	
Experimental	Calculated	Experimental	Calculated
4000	4000	4000	4000
2952	3092	2952	3092
1675	1685	1712	1737
1607	1607	1651	1683
1521	1521	1623	1655
1449	1486	1578	1578
1408	1411	1450	1487
1319	1328	1420	1452
1265	1263	1384	1417
848	844	1339	1364
50	50	1319	1319
		1148	1156
		1117	1125
		1087	1103
		1081	1089
		1062	1066
		928	932
		855	853
		50	50
